# Acute cardiovascular, autonomic and endocrine responses to a one-hour competitive esports session: a randomized controlled crossover study

**DOI:** 10.1186/s13102-026-02159-w

**Published:** 2026-10-12

**Authors:** Markus Soffner, Fabian Tomschi, Udo F. Wehmeier, Thomas Hilberg

**Affiliations:** Department of Sports Medicine, University of Wuppertal, Moritzstraße 14, Wuppertal, 42117 Germany

**Keywords:** Esports, Competitive gaming, Heart rate, Heart rate variability, Blood pressure, Stress physiology, Cortisol

## Abstract

**Background:**

Competitive esports involve prolonged sedentary behavior combined with high cognitive and emotional demands, potentially influencing acute physiological responses. However, evidence regarding acute cardiovascular, autonomic, and endocrine responses to competitive gaming remains inconsistent and methodologically heterogeneous.

**Methods:**

In this randomized controlled crossover study, sixteen healthy male EA Sports FC players completed a one-hour competitive gaming session and a control condition (documentary viewing). Heart rate (HR), root mean square of successive differences (RMSSD) and standard deviation of NN-intervals (SDNN) for heart rate variability (HRV) were recorded continuously. Peripheral and central blood pressure, pulse wave velocity (PWV) and salivary cortisol were assessed at baseline, post-session, and after 15 min of rest. Linear mixed-effects models examined the effects of condition, time, and their interaction while accounting for period and sequence effects.

**Results:**

RMSSD (*p =* 0.005, ηₚ² = 0.13) and SDNN (*p =* 0.040, ηₚ² = 0.08) showed a significant *Condition x Time* interaction, with an increase in the control condition, whereas no significant temporal changes were observed during gaming. No significant *Condition x Time* interactions were observed for HR, PWV, peripheral and central blood pressure. Salivary cortisol decreased over *time* in both conditions (*p <* 0.001, ηₚ² = 0.33) without condition-specific effects (*p =* 0.765).

**Conclusion:**

In this laboratory setting, no significant condition specific temporal changes in acute hemodynamic or cortisol responses were detected during competitive gaming compared with passive documentary viewing. The observed differences in cardiac autonomic regulation were primarily driven by an increase in HRV during the control condition, whereas no significant temporal changes in HRV were observed during gaming.

**Trial registration:**

The study was preregistered in the German Clinical Trials Registry on 05 June 2024 (ID: DRKS00034399).

**Supplementary Information:**

The online version contains supplementary material available at https://doi.org/10.1186/s13102-026-02159-w .

## Introduction

Esports, which is defined as organized competitive digital gaming, has developed into a global phenomenon, engaging millions of players worldwide across recreational, amateur, and professional levels [1, 2]. Competitive gaming is no longer a niche activity but represents a structured performance domain with organized leagues, ranking systems, and professional training environments [3]. Given the rapid growth of esports participation and viewership, esports have become increasingly relevant from a public health and sports science perspective with growing scientific interest, particularly regarding its potential health effects [4, 5].

A major driver of this interest is the predominantly sedentary nature of esports, as players typically spend prolonged periods in a seated position. Empirical studies indicate that competitive esports players spend approximately 20 h per week engaged in video game play, with a substantial proportion reporting weekly durations exceeding 30 h [6, 7]. Prolonged sedentary behavior has been identified as an independent risk factor for cardiovascular disease, type 2 diabetes, increased pain prevalence, and all-cause mortality, even after adjustment for levels of moderate-to-vigorous physical activity [8–10].

However, long periods of sitting are not the only strain in esports. It is characterized by sustained attentional load, rapid decision-making, emotional involvement, and performance pressure, particularly in ranked or tournament settings [11, 12]. These demands may elicit acute psychophysiological stress responses that differ fundamentally from passive sedentary screen-based activities such as viewing television or streams. Acute stress responses are of particular interest, as they reflect transient activations of cardiovascular, autonomic, and endocrine systems that may become relevant when occurring repeatedly or with insufficient recovery [13].

Although several studies have investigated physiological responses to esports, the available evidence remains fragmented and methodologically heterogeneous. Competitive gaming has been shown to induce elevations in heart rate (HR) and reductions in heart rate variability (HRV), including the root mean square of successive differences (RMSSD) and the standard deviation of NN-intervals (SDNN) [14, 15], whereas others report minimal or inconsistent cardiovascular and endocrine responses [16]. Overall, the current literature remains inconclusive, which may be explained by differences in game genres, gaming context (e.g., casual vs. competitive) or the different methodological approaches across studies [17].

Most existing investigations have focused on single matches or short gaming bouts, often lasting between 10 and 30 min [14, 16]. In addition, previous studies often assess isolated physiological parameters, such as HR or HRV alone, while integrated assessments combining cardiovascular, autonomic, and endocrine markers within a single experimental framework are scarce [15, 18]. Understanding the acute physiological demands of esports is essential to differentiate gaming-specific stress responses from unspecific sedentary effects and may provide a foundation for evidence-based approaches to health monitoring, training and recovery in esports players. Such knowledge is particularly relevant in performance settings characterized by prolonged cognitive stress in the absence of substantial physical activity.

Therefore, the aim of the present randomized controlled crossover study was to investigate the acute cardiovascular, cardiac autonomic, endocrine responses, and subjective cognitive fatigue of healthy male esports players to a one-hour competitive esports session in a sports simulation game compared with a control condition including passive documentary viewing. It was hypothesized that competitive gaming would elicit greater acute cardiovascular, cardiac autonomic, and endocrine responses than the passive control condition, reflected by higher HR, lower HRV, higher peripheral and central blood pressure, higher pulse wave velocity (PWV), and higher salivary cortisol.

## Materials and methods

### Ethics

The study was approved by the ethics committee of the University of Wuppertal (reference: SK/AE 230307) and conducted in accordance with the Declaration of Helsinki. Written informed consent was obtained from all participants prior to study participation. Reporting of this study follows the CONSORT 2025 checklist [19], with additional consideration of the CONSORT extension for randomized crossover trials [20]. The study was preregistered in the German Clinical Trials Registry on 05 June 2024 (DRKS00034399).

### Study design

This randomized controlled crossover study was conducted from November 2024 to September 2025 at the eSport.Hub in Solingen, Germany. All sessions were carried out in a quiet, standardized room with normal ambient lighting. To minimize external distractions, only the participant and the researcher were present in the room during the measurements. The same room setup was used for both the gaming and control conditions. Each participant had two appointments at least one week apart as a wash-out period, with both appointments taking place at the same time of the day, with all sessions taking place between 10:00 and 14:00. At the first appointment, a declaration of consent was obtained, anthropometry was taken and a questionnaire was filled out asking about demographic data, gaming behavior and physical activity. The gaming behavior questions asked about player category, gaming time, how long the participant had been playing video games and the usual length of a gaming session. For physical activity, the European Health Interview Survey – Physical Activity Questionnaire [21] and for sedentary time, the Sedentary Behavior Questionnaire [22] was used. HR and HRV were recorded over the entire measurement period.

The first series of measurements (T0) was carried out as a baseline after a 10-minute seated rest period to allow for physiological stabilization. This consisted of a subjective survey of cognitive fatigue on a scale of 0–10 (0 = not cognitively fatigued at all; 10 = cognitively exhausted), the collection of a saliva sample using a suction roller (Sarstedt), measurement of blood pressure and PWV. Then, depending on the order, either the control or the intervention was carried out.

**Fig. 1:**
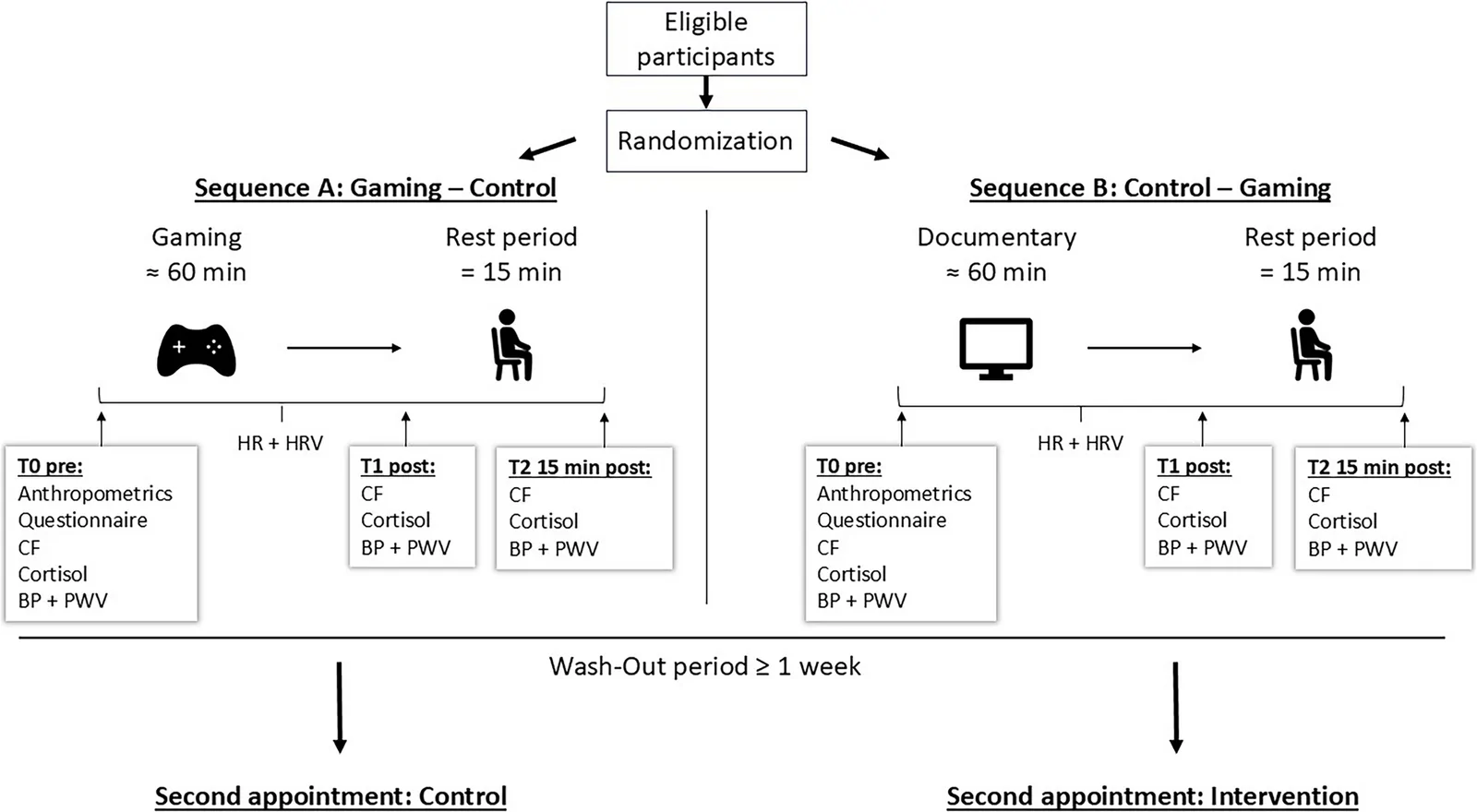
Overview of the randomized, controlled crossover study design BP = Blood pressure; HR = Heart rate; HRV = Heart rate variability; PWV = Pulse wave velocity; CF = Cognitive fatigue

Immediately afterwards, a second series of measurements (T1) was carried out and after a further 15 min of rest a third series of measurements (T2). During the entire conditions, participants remained seated. Figure 1 provides an overview of the study design. No changes to methods were made after inclusion of the first participant.

### Participants

Participants were recruited using convenience sampling via esports organizations, EA Sports FC groups on social media (*Facebook*, *Instagram*, etc.) and the University of Wuppertal through flyers and classroom presentations. Interested people received information about the content, procedure of the study as well as inclusion and exclusion criteria in advance. Healthy male participants aged 18 to 40 years, who regularly played EA Sports FC were eligible to participate in this study. Exclusion criteria included the presence of acute or chronic pain, diagnosed cardiovascular disease, the use of analgesic or cardiovascular medication and acute illness. The prerequisite was that participants should not eat anything for one hour beforehand, should not have consumed any caffeine for four hours before the measurement, and should not have engaged in any intense physical activity for 24 h beforehand. In addition, they should not have played any video games on the day of the measurement.

An a priori power analysis was conducted using GPower 3.1.9.7 [23]. based on the most comparable esports physiology study by Ketelhut and Nigg [14], who observed large partial eta-squared (ηₚ²) effect sizes ranging from 0.272 to 0.588 across multiple outcomes. Given these findings and the limited availability of comparable experimental studies, a large effect size (f = 0.4) was assumed. The power analysis was conducted for a repeated-measures ANOVA with a within-between interaction, using an alpha level of 0.05 and a statistical power of 0.8. Based on these assumptions, a minimum sample size of 12 participants was required. To consider a dropout rate of approximately 30%, 16 participants were included in this study. Accordingly, the study was primarily powered to detect large effects. Participants were randomly assigned to one of two condition orders. The random allocation sequence was generated by MS before the first appointment using an online randomization tool (GraphPad QuickCalcs, GraphPad Software, San Diego, CA, USA).

### Intervention and control condition

The intervention consisted of an approximately one-hour competitive gaming condition in the latest version of the EA Sports FC game (Electronic Arts, Redwood City, USA). As the individual games vary in length, a playing time of 50–70 min was aimed for. Participants had to play on their own accounts on a PlayStation 5 console (Sony, Tokyo, Japan) using a standard DualSense wireless controller. The game EA Sports FC is a soccer simulation and the most popular videogame in the sports simulation genre. The game involves continuous visuomotor control, rapid decision-making, tactical adjustments, offensive and defensive game situations, and emotional engagement in response to match events such as scoring, conceding goals, or losing. To maintain a competitive environment, participants could choose between the competitive modes Weekend League or Division Rivals, in which they compete online against human opponents. These modes were selected because they represent the most widely played competitive formats and reliably elicit performance-related engagement. The session consisted of continuous gameplay with brief in-game waiting periods (< 2 min) due to opponent search, reflecting realistic competitive gaming patterns. No additional applications or music were allowed during gameplay.

At the control appointment, a nature documentary (Our Planet II, Netflix, 2023) was watched instead under identical environmental settings as in the gaming condition. Participants were instructed to watch the documentary quietly and were not allowed to use smartphones or engage in any additional activities during the session. The documentary viewing condition was selected as a sedentary control to match the gaming condition with respect to sitting duration, screen exposure, and environmental conditions, while minimizing additional task-related demands. Similar screen-viewing conditions have been used in previous experimental gaming studies [24, 25]. This approach allowed physiological responses during competitive gaming to be compared with those observed during prolonged sedentary screen exposure.

### Measurements

#### Hemodynamics

All hemodynamic measurements were performed using Mobil-O-Graph (IEM, Stolberg, Germany), a validated device for the measurement of peripheral systolic and diastolic blood pressure (pSysBP, pDiaBP) [26], central systolic and diastolic blood pressure (cSysBP, cDiaBP) [27], and PWV [28]. Measurements were based on the guidelines for the management of arterial hypertension [29]. At baseline (T0), measurements were taken after the resting period of about 10 min. Participants were seated with their back supported. The cuff was placed on the left upper arm at the level of the heart with cuffs of correct size and in accordance to the participants’ circumference [29]. For the subsequent time points, no resting period was introduced to capture the acute hemodynamic response to the intervention, as done before. Measurements were repeated, if the quality of the measured arterial pressure wave was poor. The quality of the arterial pressure wave was checked automatically by the software program.

#### Cardiac autonomic function

Cardiac autonomic activity was assessed using a Polar H10 chest strap (Polar Electro Oy, Kempele, Finland) in combination with the HRV Logger application (HRV4Training, Amsterdam, Netherlands) on an iPhone [30]. The device recorded interbeat (RR) intervals continuously throughout the entire session. The Polar H10 provides high-resolution RR data and has been widely validated for HR and HRV assessment [31]. Participants were equipped with the chest strap on the sternum level, and the electrodes were moistened to ensure optimal signal quality.

The HRV Logger app recorded raw RR intervals, which were later exported in CSV format for analysis. The app’s built-in artifact correction was set to a default threshold of 25%, meaning that RR intervals deviating by more than 25% from the preceding interval were automatically excluded. Raw RR interval data were used to calculate RMSSD and SDNN for HRV parameters. HR was obtained from a separate HR export file.

RR intervals and HR were recorded continuously from baseline, throughout the entire gaming and control session, and at the two post-session time points. During the gaming condition, the timing of each match and each break was documented, allowing calculation of HR_mean_, RMSSD_mean_ and SDNN_mean_ during the active gaming period (excluding in-game breaks). During the control condition, HR_mean_, RMSSD_mean_ and SDNN_mean_ were calculated across the documentary viewing period. HR, RMSSD and SDNN values at T0, T1 and T2 were derived from time windows before and after the session.

#### Cortisol measurement

Salivary cortisol was collected at all three time points using Salivette^®^ Cortisol collection tubes (Sarstedt AG & Co., Nümbrecht, Germany). Participants placed the synthetic swab in their mouth for two minutes. After collection, samples were stored at -20 °C until processing. Saliva was extracted by centrifugation for two minutes at 1000 x g. Cortisol concentrations were determined using the Expanded Range High Sensitivity Salivary Cortisol ELISA (Salimetrics, State College, PA, USA) according to the manufacturer’s protocol. ELISA assays were measured at 450 nm and 492 nm with a BMG Fluorstar Optima reader (BMG LABTECH GmbH, Ortenberg, Germany) and the cortisol concentrations were calculated with the MARS Optima Data Analysis software version 3.01 (BMG LABTECH GmbH, Ortenberg, Germany). Two T2 samples contained insufficient saliva for analysis and were treated as missing.

### Statistical analysis

Statistical analyses were performed using R v. 4.4.2. The results are presented as means ± standard deviation. To examine the effects of condition and time on cardiovascular and stress-related outcomes, linear mixed-effects models were fitted. Condition (gaming vs. control), time (T0, T1, T2) and *Condition x Time* interaction were entered as fixed effects. To account for potential sequencing or period effects despite the counterbalanced design and one week of wash-out, both factors were additionally included as fixed factors in each model. Period represented the first versus second laboratory visit, whereas sequence represented the randomized order in which participants completed the gaming and control conditions. Participant was included as a random intercept to account for repeated measurements within individuals.

Normality was evaluated by visual inspection of Q-Q plots and histograms for all parameters. In case of a significant main effect, Bonferroni-adjusted pairwise comparisons were performed. Significant *Condition x Time* interactions were explored using Bonferroni-adjusted simple-effects comparisons, including comparisons between time point within each condition and between conditions at each time point. Pairwise comparisons are reported as estimated mean differences (MD) with 95% confidence intervals (CI). ηₚ² values were calculated to estimate the effect sizes and interpreted as small (ηₚ² = 0.014), medium (ηₚ² = 0.06) and large (ηₚ² = 0.14). Differences between the gaming and control conditions for HR_mean_, RMSSD_mean_ and SDNN_mean_ during the respective sessions were analyzed using paired-samples t-tests, with MD and 95% CIs reported. To assess the possible influence of the waiting periods between matches, a sensitivity analysis was additionally performed. The normality of the paired difference scores was assessed using the Shapiro-Wilk test. Statistical significance was set at *p <* 0.05.

## Results

### Participants

Sixteen healthy male participants completed both conditions. No adverse events were reported during either condition. Most of them (*n* = 7) were hobby gamers, followed by competitive gamers (*n* = 5) and professional gamers (*n* = 4). Participants typically played for an average of 3.3 ± 1.8 h during a gaming session. In this study, participants played for an average of 60.9 ± 7.6 min. Most (*n* = 8) completed four games during this period, followed by five games (*n* = 4). Only one participant completed three games and two completed seven games. The competitive gaming modes were played equally. The characteristics of the participants are presented in Table 1.

**Table 1:** Sample characteristics and lifestyle parameters

Characteristic	Participants (*n* = 16)
Age [years]^a^	26.1 ± 5.8
Height [m]	1.83 ± 0.07
Weight [kg]	96.7 ± 21.4
BMI [kg/m²]	29.1 ± 7.1
Sitting hours[hours/day]^b^	9.9 ± 3.2
Gaming hours [hours/week] ^a^	16.5 ± 10.3
Years of gaming [years] ^a^	16.4 ± 6.1
Physical activity [hours/week]^c^	5.1 ± 3.6
Years of regular sports participation [years] ^a^	10.9 ± 8.4

Data expressed as the mean ± standard deviation

*BMI* body mass index

^a^Assessed using self-designed questions

^b^Assessed using Sedentary Behavior Questionnaire

^c^Assessed using European Health Interview Survey – Physical Activity Questionnaire

### Hemodynamics and autonomic responses

PSysBP showed a significant main effect of *Time* (F (2, 74) = 4.70, *p =* 0.01, ηₚ² = 0.11), with a significant decrease from T0 to T2 across both conditions (MD = 6.78 mmHg, 95% CI [1.17, 12.39], *p =* 0.012). PDiaBP showed a significant main effect of *Condition* (F (1, 74) = 13.282, *p <* 0.001, ηₚ² = 0.15) with higher pDiaBP values during the gaming condition than during the control condition (MD = 4.33 mmHg, 95% CI [1.96, 6.70], *p <* 0.001). PWV showed a significant main effect of *Time* (F (2, 74) = 3.84, *p =* 0.026, ηₚ² = 0.09) with a significant decrease from T0 to T2 across both conditions (MD = 0.21 m/s, 95% CI [0.021, 0.394], *p =* 0.024).

HR decreased over *Time* in both conditions (F (2, 74) = 6.21, *p =* 0.003, ηₚ² = 0.14), with values at T0 being higher than at T1 (MD = 3.93 bpm, 95% CI [0.49, 7.36], *p =* 0.019) and T2 (MD = 4.55 bpm, 95% CI [1.12, 7.98], *p =* 0.005). There was also a significant difference in *Condition* (F (1, 74) = 9.07, *p =* 0.004, ηₚ² = 0.11) with higher values for the gaming condition (MD = 3.44 bpm, 95% CI [1.17, 5.72], *p =* 0.004). RMSSD showed a significant *Condition × Time* interaction (F (2, 74) = 5.74, *p =* 0.005, ηₚ² = 0.13). Specifically, RMSSD increased from T0 to T1 in the control condition (MD = 11.51 ms, 95% CI [2.51, 20.52], *p =* 0.008), whereas no significant changes were observed in the gaming condition. SDNN also revealed a significant *Condition × Time* interaction (F (2, 74) = 3.36, *p =* 0.040, ηₚ² = 0.08). In the control condition, SDNN increased from T0 to T1 (MD = 15.93 ms, 95% CI [5.66, 26.19], *p <* 0.001) and from T0 to T2 (MD = 10.44 ms, 95% CI [0.18, 20.71], *p =* 0.045). SDNN was lower during gaming than during the control condition at T1 (MD = 13.77 ms, 95% CI [5.42, 22.12], *p =* 0.002) and T2 (MD = 9.34 ms, 95% CI [0.99, 17.69], *p =* 0.029). However, there were no significant changes in the gaming condition. Cognitive Fatigue showed a significant main effect of *Time* (F (2, 74) = 3.26, *p =* 0.044, ηₚ² = 0.08) with a significant increase from T0 to T1 across both conditions (MD = 0.72 points, 95% CI [0.02, 1.41], *p =* 0.040). All hemodynamic and autonomic parameters at the different time points are presented in Table 2.

**Table 2:** Changes in outcomes between the gaming and control condition

Outcome	Gaming (*n* = 16)			Control (*n* = 16)			*p*-values		
	T0	T1	T2	T0	T1	T2	Time x condition	Time	condition
Peripheral systolic BP [mmHg]	137.6 ± 12.8	138.4 ± 8.7	133.1 ± 10.9	135.4 ± 11.8	130.8 ± 11.8	127.3 ± 14.1	0.842	**0.012**	0.082
Peripheral diastolic BP [mmHg]	80.6 ± 13.0	84.6 ± 13.4	81.3 ± 14.4	76.6 ± 10.3	76.8 ± 10.4	78.9 ± 10.7	0.966	0.100	**< 0.001**
Central systolic BP [mmHg]	118.3 ± 11.7	115.8 ± 13.4	113.3 ± 13.1	120.0 ± 8.3	116.3 ± 11.4	115.3 ± 13.2	0.884	**0.015**	0.304
Central diastolic BP [mmHg]	80.3 ± 12.2	79.7 ± 12.0	81.2 ± 10.5	81.3 ± 10.5	81.7 ± 11.1	83.2 ± 11.2	0.920	0.501	0.151
PWV [m/s]	5.7 ± 0.5	5.6 ± 0.6	5.6 ± 0.5	5.7 ± 0.5	5.5 ± 0.5	5.4 ± 0.6	0.301	**0.026**	0.133
Heart rate [bpm]	81.7 ± 12.1	78.7 ± 11.2	77.4 ± 11.5	79.0 ± 11.3	74.1 ± 9.4	74.2 ± 10.1	0.782	**0.003**	**0.004**
RMSSD [ms]	41.7 ± 25.3^#^	37.1 ± 21.0	36.5 ± 20.4	32.9 ± 15.8^#^	44.4 ± 17.8^T0^	41.8 ± 18.6	**0.005**	0.420	0.558
SDNN [ms]	70.4 ± 33.4	71.4 ± 27.6^#^	70.3 ± 28.6^#^	69.2 ± 32.9	85.1 ± 30.5 ^T0, #^	79.6 ± 30.5 ^T0, #^	**0.040**	**0.020**	0.004
Cognitive fatigue [0–10]	2.1 ± 2.1	2.8 ± 2.0	2.5 ± 1.9	1.9 ± 2.1	2.6 ± 1.7	2.4 ± 1.7	0.992	**0.044**	0.531

Data are expressed in mean ± standard deviation

*BP *Blood pressure, *PWV *Pulse wave velocity, *RMSSD* Root mean square of successive differences, *SDNN *Standard deviation of NN intervals

Superscript ^T0^ indicates a significant difference from T0 within the same condition (Bonferroni-corrected). A superscript ^#^ indicates a significant difference between conditions at the respective time point

Bold values indicate statistical significance (*p <* 0.05)

**Fig. 2:**
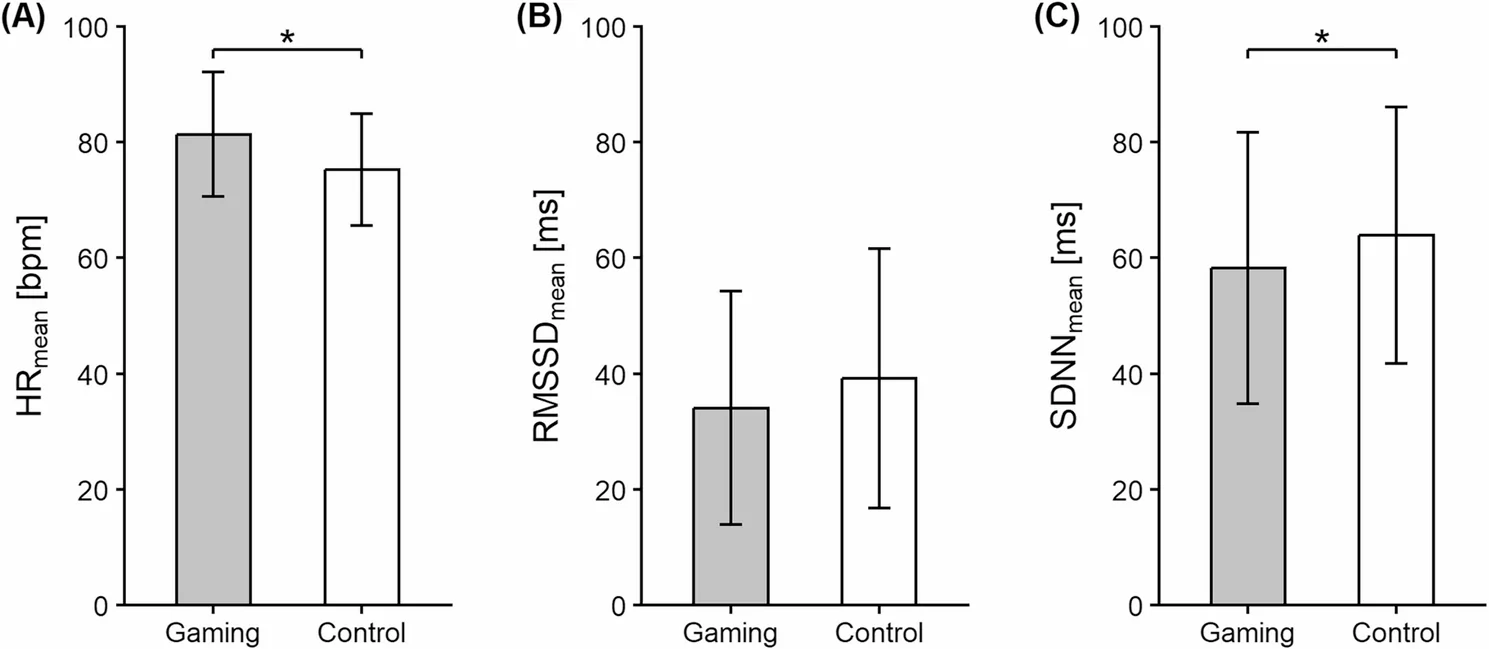
Heart rate and heart rate variability parameters during the gaming and control condition Bars represent mean ± standard deviation. HR = Heart rate; RMSSD = root mean square of successive differences; SDNN = standard deviation of NN-intervals. * *p <* 0.05

When comparing data while gaming and watching a documentary, a paired t-test revealed that HR_mean_ was significantly higher during gaming (82.5 ± 11.5 bpm) than during the control condition (MD = 7.31 bpm, 95% CI [1.55, 13.06], *p =* 0.016). In contrast, no significant difference in RMSSD_mean_ was observed between gaming and the control condition (MD = − 5.13 ms, 95% CI [-15.91, 5.65], *p =* 0.326). However, SDNN_mean_ was significantly lower during gaming compared to the control condition (MD = -10.49 ms, 95% CI [-20.57, -0.41], *p =* 0.042). These data are illustrated in Fig. 2. A sensitivity analysis including the waiting periods between matches showed comparable results with higher HR_mean_ during gaming (MD = 7.39 bpm, 95% CI [1.63, 13.14], *p =* 0.015), lower SDNN_mean_ (MD = -10.05 ms, 95% CI [-19.58, -0.53], *p =* 0.040), and no significant difference in RMSSD_mean_ (MD = -4.38 ms, 95% CI [-15.71, 6.95], *p =* 0.422).

### Cortisol measurement

**Fig. 3:**
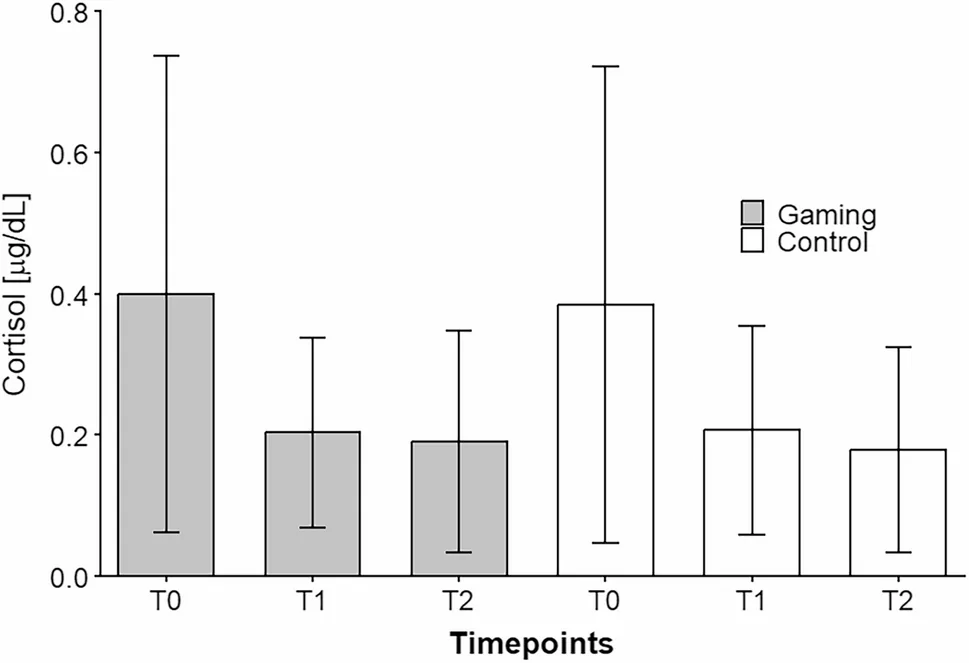
Saliva cortisol concentrations in mean ± standard deviation during the gaming and control condition At T2, *n* = 15 per condition due to insufficient saliva volume in one sample from each condition. T0 = Baseline; T1 = Post-session; T2 = 15 min post-session

The analysis revealed a significant main effect of *time* (F (2, 72.06) = 18.06, *p <* 0.001, ηₚ² = 0.33). Post hoc analyses across both conditions showed that cortisol concentrations were significantly higher at T0 than at T1 (MD = 0.187 µg/dL, 95% CI [0.091, 0.283], *p <* 0.001) and T2 (MD = 0.221 µg/dL, 95% CI [0.123, 0.319], *p <* 0.001). The results of the cortisol measurements are displayed in Fig. 3.

Significant period effects were observed for HR, RMSSD, SDNN, pDiaBP, and cortisol. HR, pDiaBP and cortisol were higher during the first period, whereas RMSSD and SDNN were lower. Significant sequence effects were observed for pDiaBP and cDiaBP. The results for these effects are provided in Supplementary Table S1.

## Discussion

### Key results

The present randomized controlled crossover study examined the acute cardiovascular, cardiac autonomic, and endocrine responses to a one-hour competitive esports session in a sports simulation game compared with a control condition. The study hypothesis was not supported. Condition-specific differences were observed for RMSSD and SDNN, but primarily driven by increases during the control condition, whereas no significant temporal changes in HRV were observed during gaming. HR_mean_ was significantly higher and SDNN_mean_ significantly lower during gaming than during the control condition. No significant *Condition x Time* interactions were observed for hemodynamic outcomes or cortisol, while cortisol decreased and cognitive fatigue increased over time irrespective of condition.

### Hemodynamic responses to competitive gaming

PSysBP decreased over time across both conditions, whereas pDiaBP was overall higher during the gaming condition than during the control condition. However, neither parameter showed significant *Condition x Time interaction*, indicating that the temporal blood pressure responses did not differ significantly between conditions. This pattern partly aligns with previous findings showing stable blood pressure during gaming and reductions during passive viewing [25], although other studies have reported increases during gaming [14, 32].

Baseline blood pressure values were relatively elevated in both conditions, potentially reflecting anticipatory arousal or insufficient recovery from prior daily demands. These elevated baseline values may have reduced the potential for further gaming-related increases. Although pDiaBP was overall higher during the gaming condition, the absence of a significant *Condition x Time* interaction indicated that this finding should not be interpreted as an acute gaming-induced increase in blood pressure.

Beyond peripheral blood pressure, central hemodynamic parameters may provide additional insight into cardiovascular load during competitive gaming. To date, central blood pressure and PWV during competitive gaming have been investigated only by Ketelhut and Nigg [14], reporting increases in both parameters across two different game genres. In contrast, no statistically significant gaming-specific changes in central blood pressure in the present study were detected, while PWV decreased over time irrespective of condition.

### Cardiac autonomic responses to competitive gaming

The clearest differences between the gaming and control conditions were observed in cardiac autonomic regulation. While HR decreased over time in both conditions without a differential temporal response, HR_mean_ remained higher during gaming, indicating an elevated cardiovascular activation. Current literature shows conflicting results in this regard. While Ketelhut et al. reported an increase in HR, some other studies reported no significant increase in HR during the game [16, 25]. One reason for this could be the competitive nature, which was primarily investigated in both this study and that of Ketelhut and Nigg [14]. Previous findings suggest that an increase in HR is not caused by the game itself, but rather by the competitive environment [32, 33].

In contrast, significant *Condition x Time* interactions in RMSSD and SDNN indicate different HRV response patterns between gaming and control conditions. Specifically, both HRV outcomes increased over time in the control condition, whereas no significant temporal changes were detected during gaming. In addition, the analysis of mean values during gaming and the control condition showed a significantly lower SDNN_mean_ during gaming. These findings are partly consistent with previous research reporting reduced HRV during gaming, interpreted as suppressed parasympathetic activity and a shift toward sympathetic dominance [14, 34]. In the present sample, the baseline values for RMSSD and SDNN were relatively low in both conditions, which, according to the literature, can occur. A systematic review by Leis and Lautenbach reported that participants in gaming studies are often already stressed upon arrival at the laboratory [17]. Consistent with this, period effects showed that HR, pDiaBP, and cortisol were higher at the first appointment, while RMSSD and SDNN were lower. This pattern may reflect greater activation or unfamiliarity with the laboratory setting during the first appointment. Because the condition order was randomized and counterbalanced, these period-related differences were not systematically linked to one experimental condition. Overall, these findings indicate different temporal response patterns regarding HRV between competitive gaming and documentary viewing, primarily driven by the increase during the control condition.

Subjective cognitive fatigue increased significantly from T0 to T1 across both conditions with no condition-specific changes. This suggests that perceived cognitive fatigue may reflect a general effect of prolonged screen-based engagement rather than competitive gaming per se.

### Endocrine responses

Cortisol levels decreased over time in both conditions from T0 to both T1 and T2, reflecting a significant temporal effect, while no significant interaction was observed. Previous findings on cortisol responses to gaming are inconsistent, with studies reporting increases [35, 36], no changes [37, 38] or decreases [16]. This variability suggests that cortisol responses in esports are highly context-dependent and not a uniform marker of stress. Moreover, cortisol secretion appears to be modulated by the subjective appraisal of a situation rather than by cognitive or emotional arousal alone [39]. Methodological factors further contribute to divergent findings. Studies reporting increases in cortisol often assessed blood cortisol or investigated highly competitive esports environments like a tournament, which may capture more immediate or pronounced cortisol responses [35, 36]. In contrast, studies relying on salivary cortisol measurements and standardized laboratory gaming protocols frequently reported unchanged or decreasing cortisol levels [16, 37]. In addition, studies examined different game genres that could also potentially have an influence and lead to different results.

In the present study, elevated baseline levels followed by a decline across both conditions may reflect anticipatory arousal related to the experimental setting and subsequent habituation. This suggests that cortisol responses may depend on the contextual framing of play, such as competitive relevance, game genre or evaluative pressure, rather than on competitive gaming itself, particularly in controlled laboratory environments. This interpretation is consistent with previous findings indicating that physiological activation alone does not necessarily elicit an increase in cortisol and that the endocrine response depends on the specific characteristics and perceived stressfulness of the task [40, 41]. Accordingly, the significant temporal decrease of cortisol should not be interpreted as evidence for the absence of psychological stress as psychological stress does not necessarily result in measurable cortisol response [42].

The experimental context should also be considered when interpreting the hemodynamic and cortisol findings. Although participants completed competitive online matches, a one-hour EA Sports FC session under controlled laboratory conditions differed from high-stakes competitive settings characterized by greater social-evaluative pressure or performance relevance. Higher-stakes, longer, or tournament-based sessions may produce different response patterns.

### Limitations

Several limitations of the present study should be acknowledged. First, the sample size was relatively small and limited to healthy male participants. This restricts the generalizability of the findings to female players, older adults, players with pre-existing health conditions and broader esports populations. The relatively high and heterogeneous BMI of the sample and the absence of an objective measure of player skill and the lack of quantification of competitive intensity and match performance may also limit generalizability and may have contributed to interindividual variability in physiological responses. Although several aspects of the gaming session were standardized, participants played two different competitive modes, completed different numbers of matches and session duration varied within the predefined 50–70 min window. In addition, the a priori sample size calculation was based on an assumed large effect size. Therefore, the study may have been underpowered to detect small-to-moderate but potentially meaningful physiological effects. Consequently, the possibility of type II error cannot be excluded and non-significant findings should not be interpreted as evidence for the absence of physiological effects. Second, the 10-minute resting periods prior to baseline assessment may not have been sufficient to ensure full physiological stabilization. Baseline cardiovascular and autonomic values may therefore have been influenced by anticipatory arousal related to the laboratory setting, the measurement procedures, or the upcoming competitive gaming task. Accordingly, the non-significant findings should be interpreted cautiously and within the context of the observed baseline state and the limited statistical power. Third, the study was conducted under controlled laboratory conditions. While this allowed for standardized assessments, the experimental setting may not fully reflect real-world competitive environments such as tournaments, which are often characterized by greater social-evaluative pressure, audience presence, team communication, financial or ranking consequences, greater emotional involvement and higher performance relevance. Therefore, the present findings cannot be generalized to high-stakes or professional esports competition. However, this setting may more closely reflect the usual way of playing at home in front of screens, where EA Sports FC players predominantly play these modes. Finally, the competitive gaming session consisted of approximately one hour of play and focused on a single esports title and genre. Therefore, the findings cannot be generalized to longer gaming durations, other game genres, or different competitive formats. Games such as first-person shooters, multiplayer online battle arena games, or strategy games may impose different cognitive, emotional, and social demands and may therefore elicit different physiological responses. Future studies should therefore compare acute physiological responses across different esports genres and competitive contexts.

## Conclusions

This randomized controlled crossover study observed different HRV responses between a one-hour session of competitive esports in a sports simulation game and passive documentary viewing under laboratory conditions. These differences were primarily driven by an increase in HRV during the control condition, whereas no significant temporal changes were detected during gaming. Although some hemodynamic parameters and cortisol changed over time or differed overall between conditions, no significant *Condition x Time* interactions were observed for these parameters. Studies with larger sample are warranted to further investigate potentially smaller physiological responses.

## Supplementary Information

Supplementary Material 1.

Supplementary Material 2.

## Data Availability

The datasets generated and/or analysed during the current study are not publicly available due to applicable data protection regulations but are available from the corresponding author on reasonable request.

## Abbreviations

ANOVA: Analysis of variance
BMI: Body mass index
BP: Blood pressure
cDiaBP: Central diastolic blood pressure
CF: Cognitive fatigue
cSysBP: Central systolic blood pressure
HR: Heart rate
HRV: Heart rate variability
pDiaBP: Peripheral diastolic blood pressure
pSysBP: Peripheral systolic blood pressure
PWV: Pulse wave velocity
RMSSD: Root mean square of successive differences
RR: R-R interval
SDNN: Standard deviation of NN intervals
