# Supplementary material for: Acute cardiovascular, autonomic and endocrine responses to a one-hour competitive esports session: a randomized controlled crossover study
Source: BMC Sports Sci Med Rehabil. 2026 Oct 12;18:484. doi: 10.1186/s13102-026-02159-w (PMC13659994; doi:10.1186/s13102-026-02159-w)
Supplement: Supplementary file 2 [file 13102_2026_2159_MOESM2_ESM.docx]

**Supplementary Table S1. Period and sequence effects from the linear mixed-effects models**

| **Outcome** | **Period** MD (95% CI) | ***p*-value** | **Sequence**  MD (95% CI) | ***p*-value** |
| --- | --- | --- | --- | --- |
| Peripheral systolic BP [mmHg] | 2.92  (-0.81; 6.64) | 0.123 | 3.04  (-6.41; 12.50) | 0.502 |
| Peripheral diastolic BP [mmHg] | 4.75  (2.38; 7.12) | **< 0.001** | 12.60 (3.23; 22.00) | **0.012** |
| Central systolic BP  [mmHg] | -0.85  (-3.53; 1.83) | 0.529 | 6.76  (-4.06; 17.60) | 0.202 |
| Central diastolic BP  [mmHg] | 1.86  (-0.44; 4.15) | 0.111 | 11.90  (2.19; 21.50) | **0.020** |
| PWV [m/s] | 0.090  (-0.04; 0.21) | 0.155 | 0.24  (-0.27; 0.75) | 0.325 |
| Heart rate [bpm] | 4.03  (1.76; 6.31) | **< 0.001** | -4.20  (-14.50; 6.06) | 0.395 |
| RMSSD [ms] | -10.70  (-14.90; -6.45) | **< 0.001** | -3.30  (-21.80; 15.20) | 0.709 |
| SDNN [ms] | -11.00  (-15.80; -6.20) | **< 0.001** | 3.28 (-27.90; 34.50) | 0.825 |
| Cognitive fatigue [0-10] | 0.10  (-0.36; 0.57) | 0.654 | 0.15  (-1.65; 1.94) | 0.864 |
| Cortisol [µg/dL] | 0.08  (0.01; 0.14) | **0.018** | -0.03 (-0.22; 0.17) | **0.791** |

Period effects represent period 1 minus period 2; sequence effects represent sequence 1 (gaming-control) minus sequence 2 (control-gaming). PWV = pulse wave velocity; RMSSD = root mean square of successive differences; SDNN = standard deviation of NN intervals.
